# Increased Serum Concentrations of TNF-Like Weak Inducer of Apoptosis Predict Higher 28-Day Mortality in Patients with Sepsis

**DOI:** 10.1155/2019/7238705

**Published:** 2019-01-10

**Authors:** Ying Guo, Manyi Ren, Lili Ge, Chao Sun, Rui Li, Cheng'en Ma, Shujian Sui

**Affiliations:** ^1^Department of Intensive Care Unit, The Second Hospital of Shandong University, Jinan 250033, China; ^2^Department of Cardiology, Qianfoshan Hospital Affiliated to Shandong University, Jinan 250014, China; ^3^Department of Cardiovascular Medicine, The Second Hospital of Shandong University, Jinan 250033, China; ^4^Central Research Laboratory, The Second Hospital of Shandong University, Jinan 250033, China; ^5^Department of Emergency, The Second Hospital of Shandong University, Jinan 250033, China

## Abstract

We performed the current study to explore potential predictive value of serum Tumor Necrosis Factor- (TNF-) like weak inducer of apoptosis (TWEAK) concentrations for 28-day mortality in patients with sepsis. Adult septic patients (age≥18 years) admitted to a general ICU between November 2016 and October 2017 were consecutively included in our prospective observational study. TWEAK concentrations were detected in septic patients and healthy controls. Dynamic changes of TWEAK concentrations between 1st day and 3rd day of admission to ICU (ΔTWEAK concentrations) were also measured. A total of 79 septic patients were included and 19 of them (24.1%) died after a follow-up period of 28 days. We identified arterial lactate, NT-proBNP, and male gender as independent factors for 28-day mortality of patients with sepsis. The serum levels of TWEAK were significantly lower in septic patients compared to controls (417.4 ± 196.7 pg/ml versus 1243.8 ± 174.3 pg/ml,* p*<0.001). We found a positive correlation between TWEAK concentrations and SOFA score (Spearman correlation coefficient 0.235,* p*=0.037). Area under the receiver operating characteristic curve (AUROC) of ΔTWEAK concentrations for 28-day mortality was 0.754 (95% CI 0.645–0.844). We also evaluated the diagnostic performance of combinative index (ΔTWEAK concentrations and lactate) and obtained an AUROC of 0.860 (95% CI 0.763-0.928). In conclusion, our study found lower TWEAK concentrations in septic patients than those in healthy controls. Furthermore, the increased TWEAK concentrations during disease process predict higher 28-day mortality in septic patients. Dynamic changes of TWEAK should be an important supplement for current prognostic markers.

## 1. Introduction

Sepsis remains a common cause for the hospitalization of patients. In view of the characteristics of rapid onset and rapid progress, it is also a common cause for the death of hospitalized patients, especially those who are critically ill [1, 2]. Although clinicians have a relatively good understanding for the pathophysiology of sepsis, they fail to prevent it from becoming a major public health concern throughout the world [1, 2].

It is crucial for clinicians to obtain early recognition and establish rational clinical procedures for septic patients. To this end, several biomarkers such as lactate and procalcitonin (PCT) have been routinely analyzed as patients admitted to intensive care unit (ICU) and recognized as reliable indicators for prognosis prediction of sepsis [3–5]. However, the predictive value of existing biomarkers is limited and it is necessary to discover new markers for clinical decision making. Furthermore, as the definition of sepsis has been updated in 2016 (sepsis-3) [1], it is also essential to reevaluate the diagnostic performances of some preexisting biomarkers.

Tumor Necrosis Factor- (TNF-) like weak inducer of apoptosis (TWEAK) is a secreted ligand in the TNF family and exists in forms of membrane-bound and soluble variant [6, 7]. The latter can be measured in serum to reflect the total levels of TWEAK pool [6]. Through binding to its receptor fibroblast growth factor-inducible 14 (Fn14), TWEAK involves many biologic effects including tissue repair-associated processes, e.g., cell proliferation, cell migration, and angiogenesis, as well as the activation of proinflammatory signaling pathways [8, 9]. The low expression of Fn14 in normal tissues and over expression when injuries occur reveals a special role for TWEAK/Fn14 pathway in inflammation [10]. In terms of the activating effect of TWEAK on proinflammatory cytokines (IL-8, IL-6,* etc.*) [11], some previous studies have evaluated the role of TWEAK in inflammation and found significantly alternated circulating TWEAK levels in some chronic inflammatory diseases such as systemic sclerosis, liver cirrhosis,* etc. *[12, 13]. Previous studies [6, 14] also tried to reveal the role of TWEAK in patients with sepsis. However, the currently available evidence is not consistent on the following issues: discrepancy of TWEAK concentrations in septic patients and healthy controls, relationship between TWEAK concentrations and other biomarkers, correlation between TWEAK concentrations and the severity of disease,* etc. *[6, 14]. Furthermore, few studies concern the role of dynamic changes of TWEAK concentrations in this population, which might be more meaningful for the prognosis of septic patients.

In the current study, we detected the concentrations of TWEAK in a prospective observational cohort and tried to explore its potential predictive value for 28-day mortality in patients with sepsis.

## 2. Materials and Methods

### 2.1. Patients

Adult septic patients (age≥18 years) admitted to a general ICU between November 2016 and October 2017 were consecutively included in our prospective observational study. According to the diagnostic criteria of sepsis 3.0 [1], sepsis was defined as life-threatening organ dysfunction (Sequential (sepsis-related) Organ Failure Assessment (SOFA) score ≥2 points) caused by a dysregulated host response to infection. Septic patients were initially treated with broad-spectrum empirical antibiotics, e.g., carbapenem, once the diagnoses were confirmed and deescalation of empirical antibiotics were adopted 3-5 days later if possible (according to microbiological culture results). We excluded patients who died within 3 days admitted to the ICU. Our study protocol followed the guidelines of the 1975 Helsinki Declaration (revised in 2008). Written informed consent was obtained from all patients prior to their inclusion to our study. The study protocol was also approved by the Ethical Committee of our Hospital.

### 2.2. Data Collection

Several baseline clinical characteristics (e.g., age, gender, temperature, blood pressure, source of infection, antibiotic application,* etc.*) were collected as patients admitted to the ICU. Liver function, creatinine, routine blood test, PCT, and C-reactive protein (CRP) were measured within 24 hours by clinical laboratory medicine center of the hospital. N-terminal pronatriuretic peptide (NT-proBNP) (VIDAS PBN2, bioMerieux, Marcy l' Etoile, France) (within 24 hours) and serum lactate (arterial blood using a blood gas analyzer, GEM Premier 3000 with iQM; Instrumentation Laboratory, Bedford, MA) (within 30 minutes) were measured in our department. SOFA score and APACHE II (Acute Physiology and Chronic Health Evaluation score) were also calculated as soon as septic patients were included.

Serial serum samples for the detection of TWEAK levels were collected within 24 hours after admission and the 3rd day (48-72 hours after admission) of admission to ICU. The serum samples were then centrifuged for 15 minutes at 2800×g and stored at −80°C for the following assay. We detected TWEAK concentrations by using a commercially available enzyme-linked immunosorbent assay (ELISA) kits from Cusabio (Wuhan, China). Proper sample dilutions were adopted when necessary (5-fold dilution).

We confirmed 28-day mortality as the primary observational end point of our study.

### 2.3. Statistical Analysis

Continuous variables were compared by using Student* t*-test or Mann-Whitney U test as appropriate. Categorical variables were compared with *χ*2 tests or Fisher exact test. Spearman rank correlation was used for bivariate correlation analysis. Multivariate logistic regression was performed by Forward LR method.* P*<0.05 was considered as statistically significance. All the above statistical analyses were conducted by using software IBM SPSS version 22.0 (IBM Corp., Armonk, NY, USA). Receiver operating characteristic (ROC) curves were generated for evaluation of diagnostic performances. Comparisons of areas under the receiver operating characteristic curve (AUROCs) were performed by using software MedCalc, version 12.7.0.0 (MedCalc Software bvba, Ostend, Belgium).

## 3. Results

### 3.1. Baseline Characteristics of the Included Septic Patients and Factors Related to 28-Day Mortality

Eighty-three patients were initially included in our cohort, of which 4 patients died within 3 days after admission to our department. In total, 79 septic patients were included in our study and 19 patients (24.1%) died after a follow-up period of 28 days. The overall mean age of study population was 66.6 ± 17.0 years and most patients were males (55/79). Pulmonary infection was the most common source of infection, which accounted for 53.2% (42 patients) of included patients. Other infectious sources included abdomen (19 patients, 24.1%), urinary system (3 patients, 3.8%), brain (2 patients, 2.5%), breast (1 patient, 1.3%), and mandibular abscess (1 patient, 1.3%). The infectious sources of other 11 patients (13.9%) were not confirmed or diagnosed as multiple organ infections without identification of the first infected viscera. Eight patients were diagnosed as septic cancer patients. The cancer type included esophageal cancer (3 patients), lung cancer (2 patients), gallbladder cancer (1 patient), carcinoma of urinary bladder (1 patient), and diffuse large b-cell lymphoma (1 patient).

The mean serum level of TWEAK at baseline was 417.4 ± 196.7 pg/ml (median 387.0 pg/ml; interquartile range (IQR) 246.0-565.0 pg/ml). In comparison with survivors, the nonsurvivors were found to be older (*p*=0.025), more males (*p*=0.031), higher APACHE II (*p*=0.010) and SOFA score (*p*=0.001), higher serum level of lactate (*p*=0.001), and NT-proBNP (*p*=0.007). Serum concentrations of TWEAK in the 3rd day of admission were also higher in nonsurvivors (*p*=0.026) (Table 1).

In order to reveal the baseline factors related to 28-day mortality, we performed multivariate logistic regression analysis in terms of age, gender, APACHE II, SOFA score, lactate, NT-proBNP, and TWEAK (Forward LR method). We identified arterial lactate (OR 1.36, 95% CI 1.09-1.70,* p*=0.006), NT-proBNP (OR 1.000, 95% CI 1.000-1.001,* p*=0.009), and male gender (OR 15.81, 95% CI 1.54-162.48,* p*=0.020) as independent factors for 28-day mortality of septic patients.

### 3.2. TWEAK Concentrations in Septic Patients and Healthy Controls

**Figure 1:**
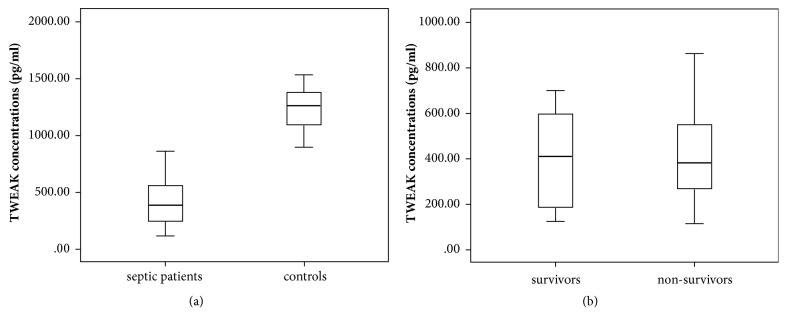
Serum TWEAK concentrations at ICU admission. (a) Serum TWEAK concentrations were significantly lower in septic patients compared to healthy controls (*p*<0.001). (b) There was no statistical significance between survivors and nonsurvivors for TWEAK concentrations (*p*=0.642).

We included 20 healthy controls (mean age 45.1 ± 11.3 years, 11 males and 9 females). The serum levels of TWEAK were significantly lower in septic patients compared to controls (417.4 ± 196.7 pg/ml versus 1243.8 ± 174.3 pg/ml,* p*<0.001) (Figure 1(a)).

### 3.3. TWEAK Concentrations and 28-Day Mortality

**Figure 2:**
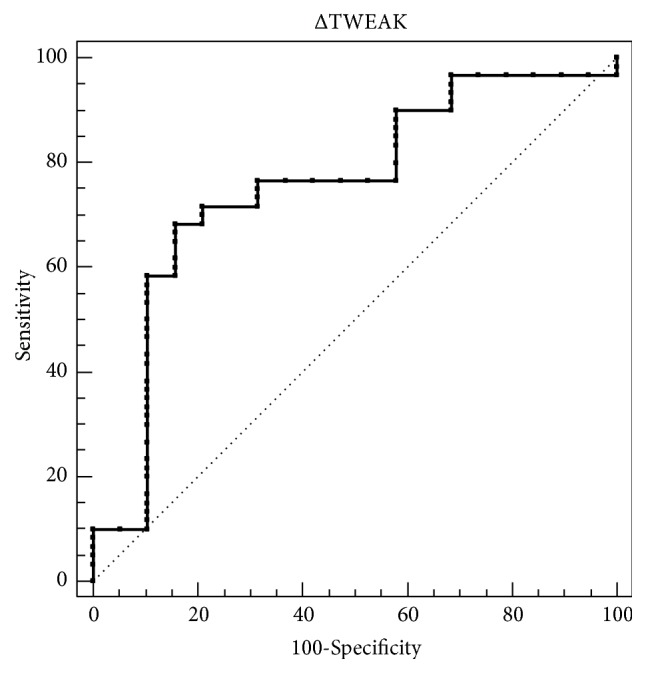
ROC curves of ΔTWEAK concentrations for 28-day mortality. ΔTWEAK concentrations refer to dynamic changes of TWEAK concentrations between 1st day and 3rd day of admission to ICU.

We found a positive correlation between TWEAK concentrations and SOFA score (Spearman correlation coefficient 0.235,* p*=0.037). However, there were no statistical significance between survivors and nonsurvivors for TWEAK concentrations (*p*=0.642) (Figure 1(b)). Considering the potential correlation between dynamic changes of TWEAK concentrations (ΔTWEAK concentrations) and 28-day mortality, we detected TWEAK concentrations in the 3rd day of admission to ICU (48-72 hours after admission). We found ΔTWEAK concentrations were significantly higher in nonsurvivors than patients survived after a follow-up period of 28-day (median 50.0 pg/ml, IQR 25.0 to 193.0 pg/ml versus median -47.5 pg/ml, IQR -184.0 to 30.5 pg/ml,* p*=0.001). We further evaluated the diagnostic performance of ΔTWEAK concentrations for 28-day mortality, which gave rise to an AUROC of 0.754 (95% CI 0.645–0.844) (Figure 2; Table 2).

**Figure 3:**
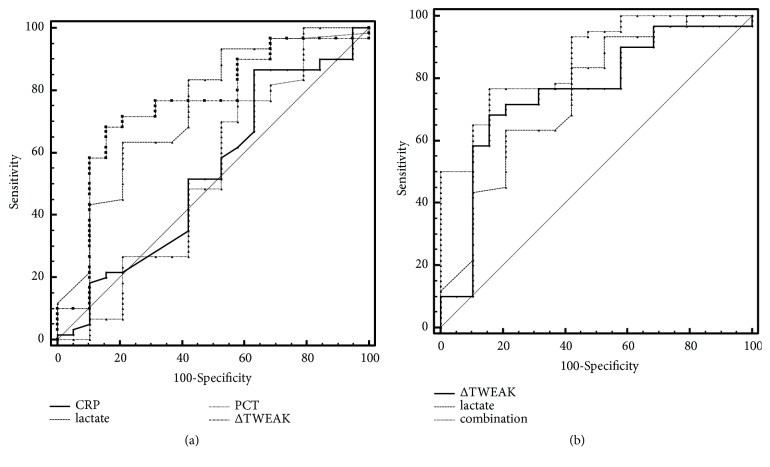
ROC curves comparing the diagnostic performances of: (a) C-reactive protein (CRP), procalcitonin, lactate, and ΔTWEAK concentrations for 28-day mortality. (b) Lactate, ΔTWEAK concentrations, and combinative index (ΔTWEAK concentrations and lactate) for 28-day mortality.

We then compared the diagnostic performance of ΔTWEAK concentrations with routine biomarkers (lactate, PCT, and CRP) for 28-day mortality. We found similar AUROCs between lactate and ΔTWEAK concentrations (0.746 versus 0.754,* p*=0.922). Besides, the diagnostic performance of ΔTWEAK concentrations was better than that of PCT or CRP (AUROC 0.754 versus 0.524,* p*=0.020 and 0.754 versus 0.534,* p*=0.024, respectively) (Figure 3(a); Table 2).

Furthermore, because of the relatively better diagnostic performance of ΔTWEAK concentrations and lactate, a combinative index was integrated by the two biomarkers through logistic regression and the AUROC was 0.860 (95% CI 0.763-0.928) (*p*=0.062 for comparing combinative index with ΔTWEAK concentrations and* p*=0.063 for comparing combinative index with lactate, respectively). The combinative index was calculated as follows: 1/(1+exp(-(3.178-0.620*∗*lactate-0.012*∗*ΔTWEAK))). A decreased combinative index predicts higher 28-day mortality in septic patients and the cut-off value was 0.7759. (Figure 3(b); Table 2).

### 3.4. Correlation of TWEAK Concentrations and Other Biomarkers

We also performed correlation analysis of TWEAK concentrations and other biomarkers. We found no correlation between TWEAK concentrations and CRP, PCT, NT-proBNP, or lactate. However, there were inverse correlations between ΔTWEAK concentrations and CRP (correlation coefficient -0.227,* p*=0.044) or PCT (correlation coefficient -0.242,* p*=0.031) (Table 3).

## 4. Discussion

In this study, we included 79 septic patients and found lower serum levels of TWEAK than healthy controls. We also explored the predictive value of increased serum TWEAK concentrations for 28-day mortality in patients with sepsis and hinted a positive correlation between TWEAK concentrations and severity of the disease.

Sepsis is a multifaceted host response to infection and has been found to activate both pro- and anti-inflammatory responses [1]. Immune imbalance is pivotal to the development of sepsis. The immune state of sepsis is diverse at different stages of the disease. Persistent and deteriorating immunosuppression during disease progress indicates a poor prognosis [15]. As a member of the TNF family, TWEAK exerts its proinflammatory effect by inducing the production of IL-6, IL-8, and monocyte chemotactic factor 1 [16]. It is therefore not surprising that TWEAK concentrations may significantly alter in the pathogenesis of sepsis.

Our study found that TWEAK concentrations were significantly lower in septic patients compared to controls, which was consistent with the conclusion of Roderburg* et al. *[6] and contrary to the result of Nagai* et al. *[14]. This discrepancy may be partially attributed to the different baseline characteristics of included patients and controls. All patients in cohort of Nagai* et al. *were diagnosed as septic shock and were treated by direct hemoperfusion; severity of disease may be an important factor for TWEAK concentrations. Meanwhile, in contrary to healthy controls, Nagai* et al. *included 20 nonseptic controls and potential alternation of TWEAK concentrations due to other pathophysiological features might be another source of discrepancy. Concentrations of TWEAK mRNA may also reveal alternations of serum TWEAK levels. Chicheportiche* et al. *[11] discovered downregulated expression of TWEAK mRNA levels in acute inflammations and this data further verified the conclusion of our study.

We found a positive correlation between TWEAK concentrations and SOFA score. As SOFA score is considered as a generally used classification system for severity of disease [17], we concluded that TWEAK concentrations were correlated to severity of the disease, which was consistent with previous studies [6, 18]. However, as mentioned above, we also found a significantly lower TWEAK concentrations in septic patients compared to healthy controls. The reason for his phenomenon remains unknown. It was said that the reason might be attributed to the diverse inflammatory response and immune state at different stages of sepsis. Further studies are required to verify this presumption. Zou* et al. *[10] found that levels of Fn14 were upregulated on pulmonary microvascular endothelial cells in murine sepsis-induced acute lung injury/acute respiratory distress syndrome. Previous studies also revealed that concentrations of CD163 increased acutely during inflammation and macrophage activation due to metalloproteinase mediated cleavage near the macrophage membrane [19]. As TWEAK activates by binding Fn14 receptor and is scavenged by binding the monocyte/macrophage scavenger receptor CD163 [8–10, 20], future studies focusing on the dynamic changes of Fn14 and CD163 in sepsis are required to reveal how the inflammatory response develops during the course of disease.

Previous studies revealed relatively higher TWEAK concentrations in nonsurvivors than survivors [6, 18]. In the current study, we found no statistical significance between survivors and nonsurvivors for TWEAK concentrations. Also, in the multivariate logistic regression analysis in terms of age, gender, APACHE II, SOFA score, lactate, NT-proBNP, and TWEAK, TWEAK concentrations were not an independent factor for 28-day mortality. However, we found that nonsurvivors displayed higher TWEAK concentrations in the 3rd day of admission compared to survivors (Table 1). Different disease course may be the source of discrepancy.

We also detected the dynamic changes of TWEAK concentrations between 1st day and 3rd day of admission to ICU (ΔTWEAK concentrations). We found ΔTWEAK concentrations were significantly higher in nonsurvivors than survivors; in other words, changes of TWEAK concentrations could predict 28-day mortality in patients with sepsis. Nagai* et al. *[14] also found that serum TWEAK levels remained stable in survivors and gradually increased in nonsurvivors in 20 patients with septic shock, which was consistent with our conclusion. Furthermore, we evaluated the diagnostic performance of ΔTWEAK concentrations for 28-day mortality in septic patients. An AUROC of 0.754 revealed a fine diagnostic performance. We also obtained an AUROC of 0.860 for combinative index (ΔTWEAK concentrations and lactate) in predicting 28-day mortality for septic patients, indicating a relatively good diagnostic performance. Therefore, dynamic changes of TWEAK should be complemented for current prognostic markers. The *p* value for diagnostic performance of combinative index versus ΔTWEAK concentrations and lactate were 0.062 and 0.063, respectively; further studies with larger cohort may be helpful for more meaningful conclusions.

We found no correlation between serum levels of TWEAK and other biomarkers. The discrepancy between our cohort and previous study [6] might be attributed to complex complications in included patients. For example, NT-proBNP may be influenced by myocardial dysfunction in patients with severe sepsis and septic shock [21]; PCT and CRP have also been evaluated as predictors for patients with surgery [22, 23]. Hence, the significance of correlation analysis between TWEAK concentrations and other biomarkers is limited in view of the complex complications in septic patients. As we have mentioned above, combinative index based on multivariate regression analyses might be feasible for this population.

It is worth mentioning the limitations of this study. First, we only detected TWEAK concentrations by using a commercially available ELISA kits. Measurements of TWEAK mRNA and Fn14 expression might be needed for further verification. Second, ΔTWEAK concentrations between admission and the 3rd day may be not enough to reflect the immune dysfunction of sepsis; more precise dynamic changes might contribute further information in the future.

## 5. Conclusions

Our study found that TWEAK concentrations in septic patients are lower than those in healthy controls. Furthermore, the increased TWEAK concentrations during disease process predict higher 28-day mortality in septic patients. Dynamic changes of TWEAK should be an important supplement for current prognostic markers. Future inquires focusing on the alternations of Fn14 and CD163 may be helpful to reveal the diverse inflammatory response and immune state at different stages of sepsis.

## Figures and Tables

**Table 1:** Characteristics of included septic patients.

	Total (n=79)	Survivors (n=60)	Non-survivors (n=19)	*P* value
Age, years	66.6 ± 17.0	64.2 ± 17.8	74.2 ± 11.6	**0.025**
Gender, male(n,%)	55 (69.6%)	38 (63.3%)	17 (89.5%)	**0.031**
MAP(mmHg)	81.0 (IQR 75.0-90.0)	82.5 (IQR 76.0-91.5)	80.0 (IQR 74.0-90.0)	0.650^#^
CRRT (n, %)	14 (17.7%)	10 (16.7%)	4 (21.1%)	0.733
Creatinine (umol/L)	100.0 (IQR 62.0-162.0)	89.75 (IQR 62.00-152.75)	134.0 (IQR 103.0-192.0)	0.072^#^
Bilirubin (umol/L)	18.0 (IQR 11.8-34.0)	18.0 (IQR 12.0-28.1)	20.0 (IQR 10.8-80.0)	0.281^#^
APACHE II	15.0 (IQR 10.0-22.0)	15.0 (IQR 9.0-18.0)	23.0 (IQR 12.0-28.0)	**0.010** ^#^
SOFA	5.0 (IQR 3.0-9.0)	4.0 (IQR 3.0-6.0)	9.0 (IQR 4.0-13.0)	**0.001** ^#^
Lactate (mmol/L)	2.0 (IQR 1.1-3.5)	1.55 (IQR 1.00-2.88)	3.5 (IQR 2.3-6.1)	**0.001** ^#^
CRP (mg/L)	154.3 ± 81.7	157.5 ± 80.0	144.2 ± 89.2	0.540
Procalcitonin (ng/ml)	5.20 (IQR 0.66-20.00)	5.20 (IQR 0.66-12.23)	4.69 (IQR 0.69-25.00)	0.757^#^
NT-proBNP (pg/ml)	1276.0 (IQR 627.0-6100.0)	1052.5 (IQR 388.5-4219.0)	6100.0 (IQR 1152.0-25000.0)	**0.007** ^#^
TWEAK at admission (pg/ml)	417.4 ± 196.7	425.1 ± 193.2	393.0 ± 210.9	0.539
TWEAK in the 3rd day of admission (pg/ml)	380.6 ± 164.1	357.7 ± 153.8	452.9 ± 178.2	**0.026**

MAP, mean arterial blood pressure. CRRT, continuous renal replacement therapy. APACHE II, Acute Physiology and Chronic Health Evaluation score. SOFA, Sequential Organ Failure Assessment. CRP, C-reactive protein. NT-proBNP, N-terminal pro-B-type natriuretic peptide. TWEAK, Tumor Necrosis Factor Weak Inducer of Apoptosis. IQR, interquartile range.

#Mann-Whitney U test

**Table 2:** Diagnostic performances of 3 routine biomarkers (lactate, NT-proBNP and CRP), ΔTWEAK and combinative index (ΔTWEAK concentrations and lactate) for prognosis of patients with sepsis.

Indicators	AUROC (95% CI)	Cut-off value	Sensitivity (%)	Specificity (%)	LR+	LR-
Lactate	0.746 (0.636-0.837)	2.2 mmol/L	63.3 (49.9-75.4)	79.0 (54.4-93.9)	3.01	0.46
procalcitonin	0.524 (0.408-0.637)	12.3 ng/ml	76.7 (64.0-86.6)	42.1 (20.3-66.5)	1.32	0.55
CRP	0.534 (0.418-0.647)	62.5 mg/L	86.7 (75.4 – 94.1)	36.8 (16.3 – 61.6)	1.37	0.36
ΔTWEAK	0.754 (0.645-0.844)	12.0 pg/ml	68.3 (55.0-79.7)	84.2 (60.4-96.6)	4.33	0.38
Combinative index	0.860 (0.763-0.928)	0.7759	76.7 (64.0-86.6)	84.2 (60.4-96.6)	4.86	0.28

CRP, C-reactive protein; TWEAK, Tumor Necrosis Factor Weak Inducer of Apoptosis; AUROC, area under the receiver operating characteristic curve; LR, likelihood ratio.

ΔTWEAK concentrations refer to dynamic changes of TWEAK concentrations between 1st day and 3rd day of admission to ICU.

**Table 3:** Correlation of TWEAK concentrations and other biomarkers.

Biomarkers	TWEAK concentrations		ΔTWEAK concentrations	
	r	*p*	r	*p*
Lactate	0.032	0.782	-0.176	0.122
CRP	0.129	0.257	-0.227	**0.044**
Procalcitonin	0.152	0.183	-0.242	**0.031**
NT-proBNP	0.082	0.475	0.052	0.647

r, Spearman correlation coefficient. CRP, C-reactive protein. NT-proBNP, N-terminal pro-B-type natriuretic peptide. TWEAK, Tumor Necrosis Factor Weak Inducer of Apoptosis.

ΔTWEAK concentrations refer to dynamic changes of TWEAK concentrations between 1st day and 3rd day of admission to ICU.

## Data Availability

The data used to support the findings of this study are available from the corresponding author upon request.
